# From bench to bedside: combining HDAC inhibitors with standard therapies in rhabdomyosarcoma treatment

**DOI:** 10.3389/fcell.2026.1774090

**Published:** 2026-02-13

**Authors:** Elisa Macrì, Marika Attili, Franco Locatelli, Francesco Marampon, Silvia Pomella

**Affiliations:** 1 Department of Hematology/Oncology, Cell and Gene Therapy, Bambino Gesù Children’s Hospital, IRCCS, Rome, Italy; 2 Department of Life Sciences and Public Health, Catholic University of the Sacred Heart, Rome, Italy; 3 Department of Radiotherapy, Policlinico Umberto I, “Sapienza” University of Rome, Rome, Italy; 4 Department of Clinical Sciences and Translational Medicine, University of Rome Tor Vergata, Rome, Italy

**Keywords:** chemo-radiotherapy sensitization, epigenetic targeting, HDAC inhibitor (histone deacetylase inhibitor), rhabdomyosarcoma, translational oncology

## Abstract

Rhabdomyosarcoma (RMS) is the most prevalent soft tissue sarcoma in children, and despite advances in multimodal therapy, progress in improving the survival of high-risk patients has been limited. Increasing evidence indicates that epigenetic dysregulation contributes to RMS pathogenesis and therapeutic resistance, particularly through aberrant activity of histone deacetylases (HDACs). HDAC inhibitors (HDACi) have shown promise in preclinical RMS models, showing enhancing of the efficacy of standard chemotherapies and radiotherapy. This mini-review summarizes recent studies exploring HDAC inhibition in combination with first-line therapies, examines the mechanistic basis for therapeutic synergy, and discusses opportunities and challenges in translating HDACi-based combinations to the clinic. By integrating mechanistic insights with translational evidence, this review outlines current progress and proposes future directions for development of HDACi-enhanced treatment strategies for this aggressive pediatric malignancy.

## 1 Epigenetic dysregulation and HDAC biology in rhabdomyosarcoma

Rhabdomyosarcoma (RMS) pathogenesis involves profound alterations in the epigenetic landscape, which sustain proliferative, metastatic, and stem-like phenotypes in both fusion-positive (FP-RMS) and fusion-negative (FN-RMS) subtypes (Gryder et al., 2017; 2019b; 2019a; 2020; Yohe et al., 2018; Marques et al., 2020; Laubscher et al., 2021; Pomella et al., 2021; 2023a; 2023b; Stanton and Pomella, 2024; Ferraro et al., 2025). Histone deacetylases (HDACs), which remove acetyl groups from histone and non-histone proteins, play a central role in shaping chromatin accessibility and transcriptional output (Seto and Yoshida, 2014). The HDAC family comprises 18 enzymes grouped into zinc-dependent Class I, II, and IV HDACs, and the NAD^+^-dependent Class III sirtuins (Seto and Yoshida, 2014; Park and Kim, 2020). RMS cells exhibit an imbalance between acetylation and deacetylation dynamics, a hallmark of epigenetic deregulation linked to impaired differentiation and sustained oncogenic transcription (Vleeshouwer-Neumann et al., 2015; Phelps et al., 2016; Gryder et al., 2019a; 2019b). While multiple HDAC isoforms are expressed in RMS, converging evidence indicates that Class I HDACs, particularly HDAC1, HDAC2, and HDAC3, are critical for maintaining the malignant state.

Indeed, Class I HDACs are highly expressed in RMS and play a central role in sustaining the malignant phenotype by coordinating cell-cycle regulation, proliferation, and survival of tumor cells (Gryder et al., 2019b). Their oncogenic impact is largely mediated through repression of tumor-suppressor genes and lineage-specific differentiation programs, together with stabilization of transcriptional states that prevent terminal myogenic differentiation (Phelps et al., 2016). Importantly, the overexpression of HDAC1, HDAC2, and HDAC3 observed in RMS is not driven by recurrent genetic alterations but rather reflects transcriptional and epigenetic reinforcement within oncogenic regulatory circuits, underscoring their role as epigenetic dependencies rather than mutational drivers (Gryder et al., 2019a; 2019b).

In FP-RMS, HDAC activity is tightly integrated into the core regulatory transcriptional networks that define tumor identity. Genome-wide chromatin analyses have demonstrated that HDACs are enriched at super-enhancer regions controlling RMS-specific oncogenic programs, where they cooperate with master transcription factors to sustain high-level, fusion-driven transcription (Gryder et al., 2019a; 2019b). HDACs directly participate in the regulation of the oncogenic fusion protein PAX3-FOXO1 (P3F), co-occupying super-enhancer regions and sustaining fusion-driven transcription (Gryder et al., 2019a; 2019b). Among Class I enzymes, HDAC3 plays a particularly prominent role in FP- and FN-RMS biology. HDAC3 is markedly overexpressed in RMS tissues compared to normal skeletal muscle and was identified as the principal HDAC restricting myogenic differentiation in a high-efficiency CRISPR-based phenotypic screen targeting Class I and II HDAC genes (Phelps et al., 2016). Mechanistically, HDAC3 functions through stable association with nuclear receptor corepressor 1 (NCOR1) and nuclear receptor corepressor 2 (NCOR2), forming the catalytically active NCOR/HDAC3 complex. This complex directly interacts with MYOD1 at regulatory regions of myogenic target genes, suppressing MYOD1-dependent transcriptional activation and thereby preventing execution of the myogenic differentiation program (Phelps et al., 2016). Consistently, genetic silencing of HDAC3 enhances H3K9 acetylation at MYOD1-bound enhancers and promoters, leading to chromatin relaxation, reactivation of myogenic gene expression, and induction of differentiation in RMS cells (Phelps et al., 2016). Beyond differentiation control, HDAC3 also contributes to the maintenance of oncogenic transcriptional programs in RMS. In FP-RMS, pharmacological or genetic inhibition of HDAC3 reduces P3F protein abundance through a regulatory cascade involving SMARCA4 downregulation, miR-27a upregulation, and subsequent destabilization of P3F mRNA (Bharathy et al., 2018). More broadly, Class I HDACs are recruited to RMS-specific regulatory regions through interactions with oncogenic and lineage-associated transcription factors, including P3F and MYOD1, as well as components of chromatin remodeling complexes such as NuRD, thereby integrating oncogenic signaling with stable epigenetic repression (Gryder et al., 2019a; 2019b; Marques et al., 2020). Post-translational regulation and signaling pathways active in RMS could further modulate HDAC stability and catalytic activity, contributing to persistent repression of differentiation-associated loci and reinforcement of transcriptional rigidity (Li and Seto, 2016).

Collectively, these regulatory layers position Class I HDACs as central nodes linking oncogenic transcription, differentiation blockade, and epigenetic stability in RMS, providing a strong biological rationale for therapeutic strategies aimed at disrupting HDAC-dependent transcriptional and chromatin regulatory networks. In this mini review, we explore the biological rationale and therapeutic potential of integrating HDAC inhibitors (HDACi) with standard first-line treatments for RMS.

## 2 HDAC inhibition in RMS: mechanistic insights and preclinical evidence

HDACi have been extensively tested in RMS models (Selim et al., 2023). Both pan-HDACi, such as Vorinostat (SAHA), Trichostatin A (TSA), OPB-801, and Belinostat (PXD-101), and selective Class I inhibitors such as Romidepsin (FK228) and Entinostat (MS-275, ENT), show antitumor activity, although FP-RMS generally displays stronger responses to HDACi, likely due to its dependency on fusion-driven transcription.

In both the RMS subtype, HDACi exert direct cytotoxic and cytostatic effects through coordinated modulation of oxidative stress, cell-cycle regulation, differentiation, and genome integrity. Across RMS subtypes, HDACi promote apoptosis by increasing intracellular reactive oxygen species (ROS), activating both intrinsic and extrinsic caspase cascades, and inducing PARP cleavage (Kutko et al., 2003; Hedrick et al., 2015; Selim et al., 2023). Elevated oxidative stress appears to be a key mediator of cytotoxicity, as antioxidant treatment reverses apoptotic effects (Chen et al., 2013; Hedrick et al., 2015; Kasiappan et al., 2019). Beyond apoptosis, HDACi exert cytostatic effects in RMS, most notably through p21-dependent cell-cycle arrest (Tarnowski et al., 2019). Intriguingly, several HDACi, including TSA, also induce myogenic differentiation, supporting their dual cytotoxic and pro-differentiation potential (Vleeshouwer-Neumann et al., 2015; Tarnowski et al., 2019).

A growing body of preclinical evidence indicates that HDACi also interfere with DNA damage response pathways and potentiate genomic instability. (Blattmann et al., 2010; Marampon et al., 2019; Cassandri et al., 2021; Rossetti et al., 2021).

Rather than representing independent phenomena, these effects can be integrated into a working mechanistic hypothesis that may account for the chemosensitizing and radiosensitizing properties of HDACi. In this proposed model, HDAC inhibition triggers a dual-hit response. First, HDACi impair DNA damage repair capacity by downregulating key components of homologous recombination (HR) and non-homologous end joining (NHEJ) repair pathways. Second, HDACi induce intracellular ROS accumulation, generating additional DNA lesions. The concomitant rise in genomic damage and suppression of repair mechanisms is hypothesized to overwhelm cellular stress responses, lower the apoptotic threshold, and ultimately enhance sensitivity to DNA-damaging agents and ionizing radiation. In parallel, HDACi-induced chromatin relaxation and transcriptional reprogramming may relieve repression of myogenic gene networks, promoting partial differentiation. While differentiation alone is insufficient for tumor eradication, it contributes to reduced transcriptional plasticity and cooperates with DNA damage–associated stress to limit tumor cell survival.

These mechanistic insights provide a compelling rationale for integrating HDACi into standard RMS treatment regimens, where their complementary actions can not only enhance therapeutic efficacy but also help overcome intrinsic and acquired resistance.

## 3 Combining HDACi with standard therapies in RMS

Current RMS treatment relies on a multimodal approach, encompassing surgery, chemotherapy, and radiotherapy. Despite these advances, survival rates remain unsatisfactory for patients with metastatic or high-risk disease, largely due to the development of chemoresistance (Chen et al., 2019). These challenges underscore the urgent need for more targeted therapeutic strategies that can minimize treatment-related toxicity while improving long-term outcomes.

### 3.1 HDACi and chemotherapy

Standard chemotherapy regimens, including Vincristine/Actinomycin-D/Cyclophosphamide (VAC) and Ifosfamide/Vincristine/Actinomycin-D (IVA), typically administered over several months in multiple cycles, remain the backbone of RMS treatment but their therapeutic effect is limited by toxicity and the development of chemoresistance (Chen et al., 2019; Zarrabi et al., 2023). Recently, HDACi have emerged as promising chemosensitizers capable of targeting pathways involved in DNA repair, apoptosis, and transcriptional maintenance.

One of the earliest studies demonstrating synergy showed that SAHA and Doxorubicin cooperatively decrease viability and increase apoptosis in FN-RMS cells, with enhanced accumulation of cells in the sub-G1 phase (Dumont et al., 2014). A subsequent study confirmed that SAHA potentiates the cytotoxicity of multiple chemotherapeutic agents, including Doxorubicin, Etoposide, Vincristine, and Cyclophosphamide. The combination with Doxorubicin was particularly effective, promoting caspase activation, downregulation of the anti-apoptotic protein Mcl-1, and increased activity of Bax and Bak, ultimately reducing colony formation in both FP- and FN-RMS cell lines (Heinicke and Fulda, 2014).

TSA has also been shown to potentiate VAC chemotherapy. By promoting terminal differentiation and enhancing drug-induced cytotoxicity, TSA may allow dose reductions without compromising therapeutic benefit (Tarnowski et al., 2019).

The Class I inhibitor ENT has generated significant interest due to its ability to reduce P3F expression. In orthotopic FP-RMS models, ENT combined with Vincristine delays tumor growth more effectively than either agent alone and demonstrates similar efficacy in patient-derived xenograft (PDX) models carrying P3F or PAX7-FOXO1 rearrangements (Bharathy et al., 2018). ENT in combination with Vincristine also reduces tumor volume and induces differentiation in FN-RMS allograft models, although PDX studies remain less conclusive due to hypersensitivity to Vincristine in some models (Bharathy et al., 2019). Furthermore, in an orthotopic allograft model of RMS treated with the chemotherapeutic agent Actinomycin D, the addition of ENT significantly delayed tumor growth compared to treatment with either agent alone or vehicle control, reinforcing its potential to enhance the efficacy of standard chemotherapy (Abraham et al., 2014).

Despite these encouraging findings, not all studies report consistent synergy between HDACi and several chemotherapeutic agents, yielding beneficial outcomes. Notably, antagonistic interactions have been reported between ENT and Vincristine in the RH18 model (FN-RMS) (Kurmasheva et al., 2019). A plausible mechanistic explanation lies in the ability of HDACi to induce p21-dependent cell-cycle arrest, which may reduce the efficacy of phase-specific agents such as vincristine that require active mitosis for maximal cytotoxicity.

These discrepancies underline the importance of optimizing dose, schedule, and RMS subtype selection. They also highlight that HDAC inhibition may protect tumor cells from selected chemotherapies under certain conditions, emphasizing the need for rational, mechanism-driven design of combination regimens rather than empirical pairing. A more recent study evaluating ENT in FP- and FN-RMS PDXs treated with chemotherapy agents employed in the relapse setting (i.e., Vinorelbine, Cyclophosphamide, Doxorubicin, Topotecan) showed that synergy is largely restricted to FP-RMS models, further highlighting subtype-specific responses (Chauhan et al., 2024). Table 1 provides a summary of the HDACi, their concentrations, the chemotherapeutic agents used in combination, and the RMS models utilized in the experimental procedures of the studies discussed in this review.

**TABLE 1:** Table summarizing HDACi tested in combination with chemotherapy agents. Doses and *in vitro* and *in vivo* RMS models have been reported.

HDACi	Class	HDACi dose	Combination agent	Combination dose	*In vitro* model	*In vivo* model	Refs
SAHA	pan	0.25–1 µM	Doxorubicin	0.025–1 µM	RD18 (FN)	NA	Dumont et al. (2014)
SAHA	pan	1–2 µM	Doxorubicin	0.05–0.25 μg/mL	RH30 (FP); RD (FN)	NA	Heinicke and Fulda (2014)
			Etoposide	5–30 μg/mL			
			VCR	0.5–1.5 nM			
			Cyclophosphamide	3–8 µM			
ENT	Class I	10 mg/kg	Actinomycin D	0.25 mg/kg	NA	U23674 FP-orthotopic allograft	Abraham et al. (2014)
ENT	Class I	5 mg/kg	VCR	1 mg/kg	NA	FP-orthotopic allograft; FP-PDX	Bharathy et al. (2018)
ENT	Class I	5 mg/kg	VCR	1 mg/kg	NA	FN-orthotopic allograft	Bharathy et al. (2019)
TSA	pan	0.5 µM	VCR	0-1000 pg	RH30 (FP); RD (FN)	NA	Tarnowski et al. (2019)
			Actinomycin D	0-0.1 µM			
			Cyclophosphamide	0-1.1 µM			
ENT	Class I	0.1 mL/10 g	VCR	1 mg/kg	NA	FP- xenografts (RH10, RH41, RH65); FN- xenograft (RH18, RH36)	Kurmasheva et al. (2019)
			Cyclophosphamide	150 mg/kg			
			Doxorubicin	0.275 mg/kg			
ENT	Class I	4 mg/kg	Vinorelbine	4 mg/kg	NA	FP- and FN-PDX	Chauhan et al. (2024)
			Cyclophosphamide	50 mg/kg			
			Doxorubicin	2.5 mg/kg			
			Topotecan	0.15 mg/kg			

Abbreviations: HDACi, HDAC inhibitors; SAHA, Vorinostat; ENT, Entinostat; VCR, Vincristine; FP, fusion positive RMS; FN, fusion negative RMS; PDX, patient-derived xenograft; NA, not available.

Taken together, these data illustrate the potential for HDACi to enhance chemotherapy efficacy but also underscore the need for meticulous preclinical modeling to define the conditions under which such combinations are most effective.

### 3.2 HDACi and radiotherapy

Radiotherapy plays a fundamental role in achieving local tumor control in RMS, yet its use is limited by significant long-term toxicity, particularly in pediatric patients. Radiosensitization strategies that selectively enhance tumor cell response to irradiation while sparing healthy tissue are therefore of substantial clinical interest. HDACi represent promising candidates due to their ability to impair DNA damage repair pathways, particularly those involved in resolving double-strand breaks (Groselj et al., 2013).

Several HDACi have demonstrated radiosensitizing potential in RMS models. Indeed, as single agent, SAHA, ENT, Belinostat and Romidepsin have been shown to increase ROS production, the accumulation of DNA double-strand breaks, as measured by elevated γH2AX foci (known marker of DNA damage), and to impair the expression or function of key repair proteins including RAD51 and Ku70/Ku80, suggesting compromised HR and NHEJ repair pathways. SAHA was among the first HDACi tested in this context: pretreatment decreases clonogenic survival after irradiation and increases G2/M arrest in FN-RMS cells (Blattmann et al., 2010). Additionally, SAHA reduces RAD51 and Ku80 expression after irradiation, indicating impaired activation of homologous recombination and non-homologous end joining. Interestingly, radiosensitization occurred without significant induction of apoptosis, suggesting that defects in DNA repair rather than increased cell death underlie its activity (Blattmann et al., 2010).

Similarly, Belinostat exhibits potent radiosensitizing effects in both FP- and FN-RMS. *In vitro*, it promotes G2/M cell-cycle arrest, increases ROS accumulation, and suppresses c-Myc expression. These effects impair the resolution of radiation-induced DNA damage, leading to enhanced cytotoxicity. Furthermore, *in vivo* studies demonstrate significant reductions in tumor volume and weight when Belinostat is combined with radiotherapy compared to radiotherapy alone (Marampon et al., 2019).

Romidepsin, despite limited antiproliferative activity as a single agent, increases ROS and induces DNA damage selectively in FP-RMS models, resulting in marked radiosensitization *in vitro* and *in vivo* (Rossetti et al., 2021). These results suggest subtype-specific enhancement of radiosensitivity mechanisms and identify FP-RMS as a particularly promising context for Class I HDAC inhibition.

ENT also enhances RMS radiosensitivity by reducing Cyclin A/B/D1 expression, increasing p21, promoting γH2AX accumulation, reducing ATM activation, and increasing ROS levels (Cassandri et al., 2021). ENT completely prevents tumor formation in FP-RMS xenograft models when combined with radiotherapy and substantially slows tumor progression in FN-RMS models (Cassandri et al., 2021). A subsequent work demonstrated that HDAC3 is a key mediator of radioresistance in FP-RMS. Indeed, HDAC3 silencing increases ionizing radiation-induced DNA damage, decreases RAD51 expression and ATM phosphorylation, and triggers apoptosis following irradiation. These findings were recapitulated using a selective HDAC3i, MC4448, which represents a step toward translational development of isoform-specific radiosensitizers (Cassandri et al., 2024). Table 2 summarizes the studies discussed in this review in which HDACi were used in combination with radiotherapy, including the experimental models and drug concentrations employed.

**TABLE 2:** Table summarizing HDACi tested in combination with IR. Doses and *in vitro* and *in vivo* RMS models have been reported.

HDACi	Class	*In vitro* dose	*In vivo* dose	IR dose	*In vitro* model	*In vivo* model	Refs
SAHA	pan	0-1 µM	NA	6 Gy	A-204, RD (FN)	NA	Blattmann et al. (2010)
Belinostat	pan	0.41 µM (RD); 0.23 µM (RH30)	40 mg/kg	4 Gy *in vitro,*12 Gy *in vivo* (2 Gy × 6 days)	RD (FN) and RH30 (FP)	FP- and FN-xenograft (RD, RH30)	Marampon et al. (2019)
Romidepsin	Class I	1.4 nM (RD); 0.6 nM (RH30)	1.2 mg/kg	4 Gy *in vitro*, 10 Gy *in vivo* (2 Gy × 5 days)	RD (FN) and RH30 (FP)	FP-xenograft (RH30)	Rossetti et al. (2021)
ENT	Class I	1 µM (RD); 1.9 µM (RH30)	2.5 mg/kg	4 Gy *in vitro*, 6 Gy *in vivo* (2 Gy × 3 days)	RD (FN) and RH30 (FP)	FP-xenograft (RH30)	Cassandri et al. (2021)
MC4448	HDAC3 selective	40 nM (RH30); 58 nM (RH4)	NA	6 Gy	RH4, RH30 (FP)	NA	Cassandri et al. (2024)

Abbreviations: HDACi, HDAC inhibitors; IR, ionizing radiation; SAHA, Vorinostat; ENT, Entinostat; FP, fusion positive RMS; FN, fusion negative RMS; Gy, Gray; NA, not available.

Together, these studies highlight HDAC inhibition as an attractive strategy to increase radiosensitivity in RMS, particularly in FP-RMS where standard treatments often fail to achieve adequate local control.

## 4 Conclusion and translational perspectives

HDACi have already entered testing in combination with both chemotherapy and radiotherapy in phase I-II clinical trials across a range of solid tumors and hematologic malignancies, underscoring the translational potential of the strategies outlined above (Sullivan et al., 2022). Early-phase trials have evaluated or are evaluating combinations of HDACi with radiotherapy, including studies of vorinostat or valproic acid administered concurrently with radiation in glioma, pancreatic cancer, non-small cell lung cancer, and other solid tumors to assess safety, tolerability, and radiosensitization effects (NCT00838929; NCT00313664).

Likewise, HDACi have been combined with chemotherapeutic regimens in clinical settings. Early phase I/II studies have tested Vorinostat or Abexinostat in combination with DNA-damaging agents including Doxorubicin or with multi-agent chemotherapy backbones for adult advanced sarcoma [NCT01027910 (Choy et al., 2015)].

Collectively, these clinical efforts reinforce the translational rationale that HDACi can modulate therapeutic response and resistance mechanisms to enhance the efficacy and potentially reduce the toxicity of chemotherapy and radiotherapy. Such data from ongoing and completed trials in other cancer types offer a valuable framework for designing RMS-specific combination studies.

The collective body of preclinical evidence in this mini review strongly supports the integration of HDACi into RMS treatment strategies. HDAC deregulation contributes to RMS pathogenesis by sustaining oncogenic transcriptional programs, impairing differentiation, and promoting survival pathways. HDAC inhibition disrupts these processes and enhances the efficacy of both chemotherapy and radiotherapy through increased ROS production, impaired DNA damage repair, reinforced apoptotic programs, and modulation of cell-cycle dynamics.

From a translational perspective, emerging data suggest that FP-RMS represent the subtype most likely to benefit from HDACi-based combinations possibly due to its dependence on P3F-driven transcriptional circuits, which correlate with sensitivity to HDAC inhibition (Gryder et al., 2019b; 2019a). Moreover, FP-RMS cells exhibit a selective dependency on Class I HDACs, particularly HDAC3, and targeting HDAC3 enhances DNA damage accumulation and sensitizes cells to chemotherapy and radiotherapy (Bharathy et al., 2019; Gryder et al., 2019b; 2019a; Cassandri et al., 2024). Moreover, the development of more selective HDACi, including HDAC3-specific compounds may offer a path to reduce chemotherapy or radiation dose intensity, thus minimizing long-term toxicity in pediatric patients while preserving or even enhancing antitumor efficacy.

Translation to the clinic will require rigorous optimization of dosing schedules, clarification of subtype-specific sensitivities, and the identification of predictive biomarkers. Indeed, despite preclinical insights, no defined expression threshold or clinically validated molecular signature exists to predict response to HDACi in RMS. Studies in other tumor types suggest potential predictive biomarkers, including RAD23 homolog B (HR23B) expression in cutaneous T-cell lymphoma, and HDACi-related transcriptional signatures in preclinical models, though none are yet clinically validated (Treppendahl et al., 2014). Prospective studies integrating molecular profiling, such as P3F expression levels, HDAC3 dependency, or ROS- and DNA-damage related molecular signatures, with pharmacologic response will be essential to validate such biomarkers and enable patient stratification in future HDACi-based trials.

Overall, HDACi hold significant potential to improve the therapeutic landscape of RMS. By bridging mechanistic insights with translational research, future studies may refine HDACi-based combination strategies and bring forward more effective, less toxic treatments for children with this challenging disease.
